# Theory of nodal *s*^±^-wave pairing symmetry in the Pu-based 115 superconductor family

**DOI:** 10.1038/srep08632

**Published:** 2015-02-27

**Authors:** Tanmoy Das, Jian-Xin Zhu, Matthias J. Graf

**Affiliations:** 1 Theoretical Division, Los Alamos National Laboratory, Los Alamos, NM 87545, USA; 2 Center for Integrated Nanotechnologies, Los Alamos National Laboratory, Los Alamos, NM 87545, USA

## Abstract

The spin-fluctuation mechanism of superconductivity usually results in the presence of gapless or nodal quasiparticle states in the excitation spectrum. Nodal quasiparticle states are well established in copper-oxide, and heavy-fermion superconductors, but not in iron-based superconductors. Here, we study the pairing symmetry and mechanism of a new class of plutonium-based high-T_c_ superconductors and predict the presence of a nodal *s*^+−^ wave pairing symmetry in this family. Starting from a density-functional theory (DFT) based electronic structure calculation we predict several three-dimensional (3D) Fermi surfaces in this 115 superconductor family. We identify the dominant Fermi surface “hot-spots” in the inter-band scattering channel, which are aligned along the wavevector Q = (*π*, *π*, *π*), where degeneracy could induce sign-reversal of the pairing symmetry. Our calculation demonstrates that the *s*^+−^ wave pairing strength is stronger than the previously thought *d*-wave pairing; and more importantly, this pairing state allows for the existence of nodal quasiparticles. Finally, we predict the shape of the momentum- and energy-dependent magnetic resonance spectrum for the identification of this pairing symmetry.

The unconventional mechanism of Cooper pairing is often attributed to a momentum dependent superconducting (SC) gap structure with sign reversal of its amplitude, which renders the electron pair formation in a repulsive potential background^1^. This scenario is primarily supported by the proximity of superconductivity to magnetism, and a correspondence between the energy of the magnetic resonance mode and the SC transition temperature (*T_c_*)^2^. The recently discovered 5*f*-based intermetallic actinides Pu*MT*_5_ (*M* = Co, Rh, and *T* = In, Ga) or in short Pu-115 represent a rather exotic class of superconductors, which exhibit evidence of nodal superconductivity despite the absence of a magnetic instability. However, the nuclear magnetic response (NMR)^3^,^4^,^5^ and muon spin rotation (*μ*SR) measurements^6^,^7^, later on supported by theoretical study^8^, demonstrate that spin fluctuations are significantly large, but do not drive the system into a magnetic ground state at low temperatures^6^. Therefore, a novel materials specific theoretical route to link spin fluctuations to superconductivity would be helpful in order to test whether there is a universal magnetic mechanism of superconductivity in all families of high-*T_c_* superconductors^9^.

The intermetallic Pu-115 actinide superconductors represent the link in the sequence going from the heavy-fermions (*T_c_* ~ 1 K), to the iron-pnictides (*T_c_* ~ 50 K), on to the cuprates (*T_c_* ~ 100 K), with respect to the values of the SC transition temperature *T_c_*, spin fluctuation temperature *T_s_*, as well as mass enhancement^3^,^4^. PuCoGa_5_ possesses an electron mass renormalization of m*/*m_b_* ~ 3.5 (where *m_b_* is the DFT-deduced band mass), which is many times smaller than that of the heavy-fermion superconductor CeCoIn_5_ with m*/*m_b_* ~ 30^10^,^11^. Such a moderate mass enhancement by electron correlations is well captured by dressing of 5*f*-states via the spin-fluctuation coupling of electron-hole excitations^8^,^12^. The exchange of spin fluctuations divides the electronic states into renormalized itinerant quasiparticles near the Fermi level and strongly localized incoherent states at higher binding energies. This scenario of dynamical correlation effects is consistent with the corresponding peak-dip-hump feature measured in photoemission spectroscopy^13^. The bulk Curie-Weiss susceptibility observed in the normal state of PuCoGa_5_^14^ was initially interpreted to arise from static moments^6^,^15^ but more recently suggested to come from the fluctuation of spins or valences of Pu between 5*f*^5^ and 5*f*^6^ configurations of the ground state^16^,^17^, as in some heavy-fermion superconductors^18^. Taken together, Pu-115 compounds reside in between localized and itinerant systems, for which the DFT band structure with proper mass renormalization may be the appropriate starting point to describe the low-energy spectrum, from which superconductivity emerges.

Our main finding of the DFT-based calculations of the pairing symmetry, due to a Fermi surface (FS) instability, is that a nodal *s*^±^-wave pairing symmetry is favored over the previously thought 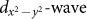 pairing symmetry. Despite the presence of four different FS pieces, the leading pairing instability arises from the enhanced scattering between hole pockets at the Γ point and electron pockets at the A = (*π*, *π*, *π*) point in the Brillouin zone. This FS topology acquires an analogy to the electronic states of the more recently discovered iron-based superconductors. However, unlike in the latter family, here the *s*^±^-wave pairing is significantly anisotropic due to nearest-neighbor electron pairing, and its nodal planes intercept the hole-like FS near the zone boundary. We further present DFT-based results of the magnetic excitation spectrum for both pairing symmetries, and observe a prominent collective spin-1 mode, which is localized in both energy and momentum within the SC phase for the *s*^±^-wave rather than for the *d*-wave symmetry. Our result of a nodal pairing state is consistent with power-law signatures in the spin-lattice relaxation rate^3^,^4^,^5^, superfluid density^6^,^7^, and zero-bias conductance peak in point-contact spectroscopy (PCS)^19^, among others. Although these results are often taken to be consistent with the assumption of a nodal *d*-wave pairing, it should be noted that these probes are only sensitive to the nodal states, not to their location on the FS. To clarify this issue we carry out a multiband PCS calculation using both nodal pairing symmetries. We find that a zero-bias conductance peak is generated in both cases, and that the PCS experimental data^19^ can well be reproduced by the nodal *s*^±^-wave pairing. Finally, the spin-fluctuation mediated pairing symmetry study provides a microscopic explanation of the pairing mechanism and order parameter symmetry in this less-explored plutonium-based family of superconductors. Such a study is needed because the existence of the sign-reversal *s*^±^-wave pairing symmetry without a node can be difficult to distinguish from the *s*^++^-wave pairing symmetry, as is the case in the iron pnictides^20^, unless the sign of the order parameter can be directly measured. On the other hand, the existence of nodes in the 115 compounds may help establish that an unusual nodal *s*^±^-wave pairing symmetry indeed exists.

## Results

### Fermi Surface Nesting and Hot-Spots

**Figure 1:**
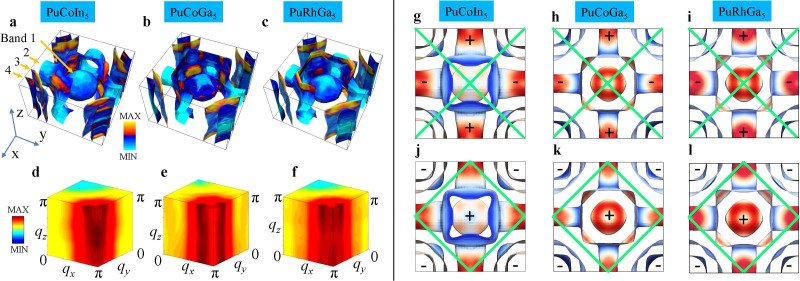
Fermi surfaces and *hot-spots* from first-principles electronic band structure calculations. (a)–(c) FS topologies in the momentum space of the Brillouin zone for all three Pu-115 materials, plotted separately in three columns. An intensity colormap is used to depict the values of the JDOS on the FSs at **Q** = (*π*, *π*, *π*). For better visualization a quadrant of the holelike FS of band 2 was clipped. The JDOS gives a qualitative estimate of the static susceptibility (Eq. (1)) for nesting vector **Q**. These images help identify the strongest nesting between bands 1 and 2 (in the vicinity of the *k_z_* = 0-plane) to bands 3 and 4 (near the *k_z_* = *π*-plane). (d)–(f) The full momentum (**q**) dependence of the static bare bubble susceptibility *χ*(**q**, *ω* = 0) is visualized in three-dimensional volume rendering. The highest intensity (red color) is in the vicinity of **q** ~ (*π*, *π*, *q_z_*). (g)–(i) Top views of FSs (same as in Fig. 1a–c) with corresponding colormaps of the magnitude of the Fermi velocities (or inverse normal-state density of states) from low (blue) to high (red). The green solid lines denote the nodal planes of the SC 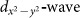 pairing symmetry. (j)–(l) Same as above, but now the green solid lines denote the nodal planes of the SC *s*^±^-wave pairing symmetry. Note, only the holelike FS of band 2 has nodes in the gap function on the cross-arms near the zone boundary.

We begin by evaluating the nature of enhanced FS scattering or *hot-spots*, and the electronic fingerprints of *s*^±^-, and 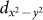-wave pairing symmetries for three known Pu-115 superconductors PuCoIn_5_ (*T_c_* = 2.5 K)^21^, PuCoGa_5_ (*T_c_* = 18.5 K)^14^, and PuRhGa_5_ (*T_c_* = 8 K)^22^. The low-energy electronic states of these compounds consist of four pairs of spin-orbit split energy bands cut by the Fermi level, as shown in Fig. 1a–c^8^,^23^. We note that these results are in agreement with similar electronic structure calculations performed independently by other groups^17^,^24^,^25^,^26^. We estimate the strength of the band-dependent scattering enhancement by computing the bare bubble two-particle response function from first-principles band structure as
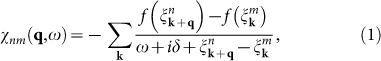
where 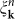 is the DFT-derived Bloch dispersion with wavevector **k** and band index *n*, and 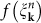 is the corresponding fermion occupation number. Figure 1d–f shows the computed static susceptibilities in a colormap plot in the three-dimensional momentum transfer **q** space. The location of the maximum of 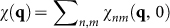 is primarily in the vicinity of **Q** ~ (*π*, *π*, *π*), with additional weights spread all along *q_z_*. This suggests that the dominant FS instability occurs between the FSs separated by **Q** in the Brillouin zone. For this value of **Q**, we identify the locations of the electronic hot-spots or the highest joint-density of states (JDOS), which satisfy 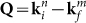, where 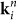 and 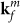 are the Fermi momenta in the initial and final states of bands *n* and *m*, respectively. The hot-spots are superimposed on the FSs using an intensity colormap as shown in Figs. 1a–c. The intensity is determined from the approximate 
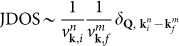
, where 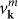 is the Fermi speed in band *m*. We immediately see a consistent scenario for all three materials, namely that the hot-spots connect bands 1 or 2 near the plane with *k_z_* = 0 to bands 3 or 4 lying in the plane with *k_z_* = ±*π*. The locations of the hot-spots dictate a pairing symmetry, which favors sign reversal for **Q**. In Fig. 1g–l the same FS topologies are shown in top view with nodal lines for 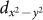-wave (top row) and *s*^±^-wave (bottom row) pairing symmetries superimposed by green solid lines. Based on the correspondence between topology of the FSs and the dominant hot-spot nesting vector, it is now possible to conjecture that the Pu-115 system may favor *s*^±^-wave pairing. Wang et al.^27^ attained very similar results for *χ_nm_*(**q**, 0), however, they emphasized the nesting at **Q** ~ (*π*, *π*, 0).

### Electron Dispersions and Density of States

Next we delineate the origin of the nodal state and compare the associated nodal electronic fingerprints for both *s*^±^-wave and 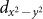-wave pairing states. We have also studied other pairing symmetries such as *d_xy_*- and 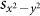-wave that are possible for the tetragonal point-group symmetry, and found that they have significantly weaker strength compared to the former two and thus are not further discussed here. Both *s*^±^- and 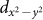-wave symmetries are essentially nearest-neighbor pairing but differ by an invariant or broken *C*_4_-symmetry, respectively, which governs the basis functions of the SC order parameters 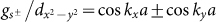. The nodal planes of the two pairing states are thus oriented along the 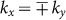-directions as shown in Fig. 1g–l, and they cut through the large squarish FS (band 2) for *s*^±^-wave pairing, while they intercept with all FSs for 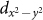-wave pairing.

**Figure 2:**
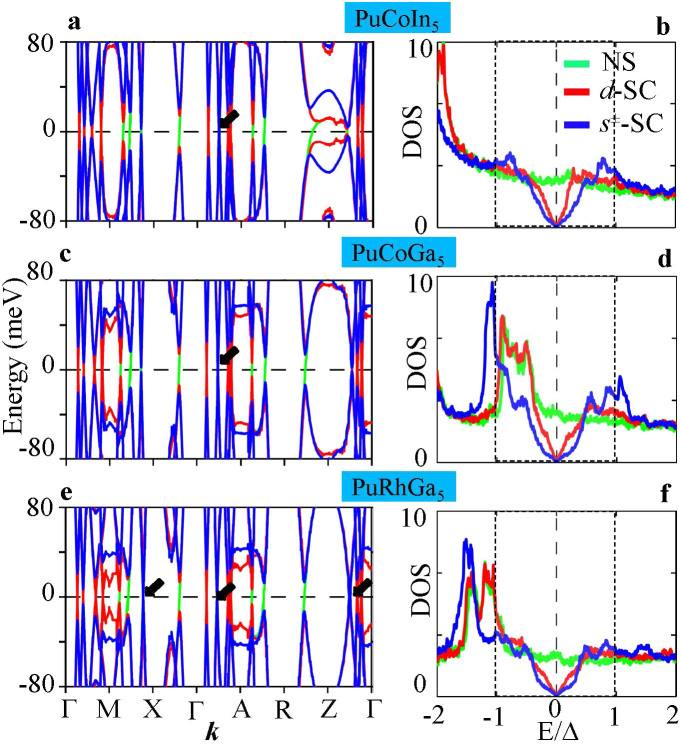
Gapped electronic DFT dispersions, nodal SC quasiparticle states. (a), (c), and (e) Low-energy electronic dispersions in the normal state with 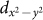
- and *s*^±^-wave gaps plotted along representative high-symmetry directions in the Brillouin zone. Here symbols Γ (Z) = (0, 0, 0/*π*), M (A) = (*π*, *π*, 0/*π*), and X (R) = (0, *π*, 0/*π*). Black arrows mark the locations of the zero-gap or nodal lines for the *s*^±^, while those for *d* wave are ubiquitous along all Γ–M, and Γ-Z directions. (b), (d), and (f) Corresponding DOS in the SC state for all three cases discussed in the corresponding left column. Residual nodal states below the SC gap are evident for both pairing symmetries. For ease of comparison an artificial gap amplitude of Δ = 40 meV (shaded region) is used in all bands and for all compounds.

To be specific, we draw the SC quasiparticle bands 
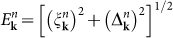
, where *n* is the band index, 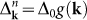, and Δ_0_ is the gap amplitude, taken to be the same for all bands and pairing states for direct comparison. Since at the FS each **k** point is uniquely associated with a specific band, *g*(**k**) carries an implicit band index *n* when solving the gap equations. While the gap amplitude is measured to be around 5–10 meV^3^,^19^, we used an artificially large value of 40 meV for all systems for better visualization. The results are compared in Fig. 2 (left column) for non-SC band (green), *d*-wave (red) and *s*^±^-wave (blue) pairing along representative high-symmetry momentum directions. We see that gapless quasiparticles evidently occur along the Γ-M direction for *d*-wave pairing, while a robust node is visible for *s*^±^-wave pairing along the Γ-A-direction for all three systems (with some accidental nodes along other directions in PuCoGa_5_). The corresponding density of states (DOS), plotted in Fig. 2 (right column) gives further insight into the energetics of the two pairing states. For the same gap amplitude, we detect that the *s*^±^-wave pairing has a larger effective gap Δ**_k_**, i.e., lower DOS inside the gap, and yields a very much ‘U’-shaped DOS with nodes at the Fermi level, in contrast to the prototypical ‘V’-shaped DOS for the *d*-wave pairing. The nodal electronic states of both pairing symmetries are evident in the NMR^3^,^4^, *μ*SR^6^,^7^, and PCS data^19^, although in these measurements the pairing symmetry has been generally interpreted to be consistent with the single-band *d*-wave pairing symmetry. We anticipate that direct spectroscopies such as angle-resolved photoemission spectroscopy (ARPES), field-angle dependent thermodynamic measurements^29^, and scanning tunneling microscopy and spectroscopy (STM/S) will be able to distinguish between these two pairing symmetries.

### Normal State Instability and Pairing Strength

We now carry out calculations of the pairing symmetry and pairing strength based on the spin-fluctuation mechanism of electron pairs in the spin-singlet channel. The methodology of the corresponding calculation is well established for other materials, and we generalize it to be combined with the DFT framework, in which all band structure information such as crystal field splitting, spin-orbit coupling are incorporated in the electronic dispersions. The dominant many-body interactions are onsite Coulomb repulsion for intra-band and inter-band components, which play separate roles for different pairing channels. These are included within the random-phase approximation (RPA). For this building block, all the relevant energetics, coming from the single-particle terms as well as many-body interactions, are incorporated within the multiband anisotropic spin 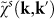 and charge 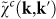 susceptibilities defined as 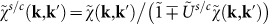, where 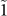 is the unity matrix, 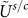 are the corresponding onsite interaction matrices (defined below), and 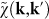 is the bare interaction defined in Eq. (1) above. All variables with tilde are of matrix dimension 16 × 16. The corresponding spin-singlet pairing matrix is^28^,^30^
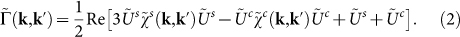


**Figure 3:**
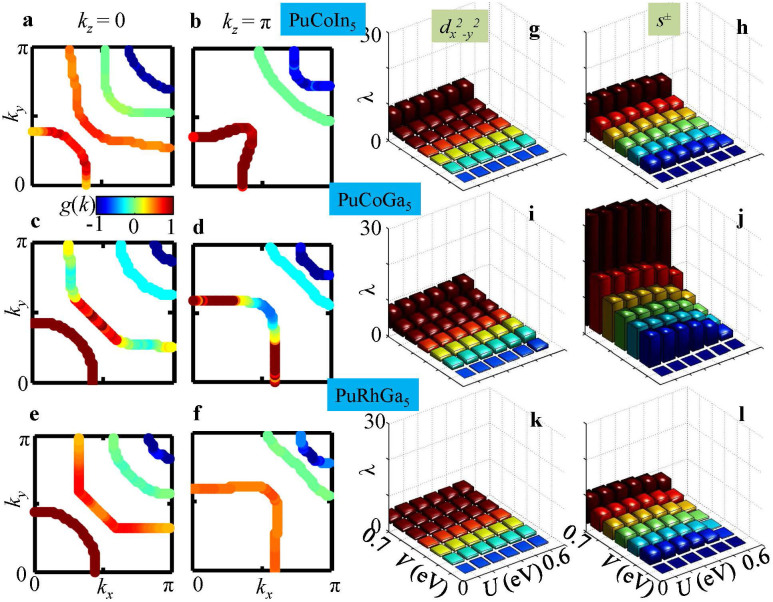
Pairing eigenfunction and total SC pairing strength *λ*. (a)-(f), The computed gap function in a color gradient plot overlayed on the corresponding FSs at two representative *k_z_* cuts, computed from Eq. (3). (g)–(l) Projected total pairing strength *λ* = Σ*_nm_λ_nm_* evaluated from Eq. (4) as a function of *U* and *V*. The height of squarish bar represents the value of the total pairing strength. Each row describes a different compound, while different columns separate different pairing symmetries. Different colors distinguish same *V* rows.

Earlier model calculations of this pairing potential in cuprates^1^,^31^, heavy-fermion systems^30^, organic superconductors^32^, and pnictides^33^, have produced good estimates of the pairing strength and pairing symmetry, consistent with corresponding experimental data. Following the same strategy, we solve the linearized multiband gap equations by the pairing eigenvalue problem as
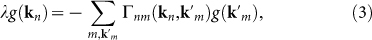
where **k***_n_* is the momentum for the *n*^th^ band and so on, and Γ*_nm_* are the components of the pair vertex in Eq. (2), after projected into the corresponding band basis. The eigenvalue calculation is performed over the entire three-dimensional FSs to estimate the dominant eigenvalue *λ*, and the corresponding eigenvector gives the leading pairing symmetry *g*(**k**) (see supplementary materials for the method of calculation). Equation (3) is solved for the representative values of intraband interaction *U* = 0.5 eV and interband interaction *V* = 0.5 eV, which yield maximal eigenvalues *λ* = 2.3, 3.5 and 2.5 for all three systems in the order discussed, and the corresponding pairing eigenfunctions are plotted in Fig. 3a–f in a blue to red colormap at the Fermi momenta for two representative *k_z_* cuts (the results are also consistent with other *k_z_* cuts and interaction values). The first point to notice is that there is no clear four-fold symmetry breaking in *g*(**k**), which excludes the presence of any significant *d*-wave pairing component. In the supplementary material, we give a detailed fit of the computed *g*(**k**) with an *s*^±^-wave gap function including higher harmonics. Our result indicates that the *k_z_* dependence of *g*(**k**) is weak, and also the presence of second and higher harmonics is negligible. Furthermore, we find the expected result that the gap anisotropy is largest in band 2, and then reduces gradually in bands 1, 4 and 3. This is expected from the *s*^±^-wave pairing symmetry as the gap maxima lie at the Γ and M points with opposite signs.

Next we study the relative strength of various possible pairing channels, and the contributions from each band. We introduce a dimensionless pairing strength by projecting Eq. (3) onto a gap function *g_α_*(**k**) with given *s*^±^-wave or 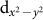-wave symmetry (denoted by *α*):
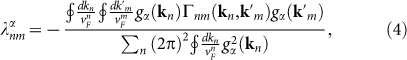
with its total value being 
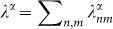
. Here 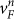 is the Fermi velocity at momentum *k_n_*. The physical interpretation of *λ_nm_* can be gained qualitatively by studying the peaks in Γ*_nm_*(**k**, **k**′). The dominant pairing potential Γ*_nm_*(**k**, **k**′) is governed mainly by the peaks of *χ*(**q**, *ω* = 0), see Fig. 1 at **q** = **k** − **k**′, because they stabilize the gap function that satisfies the condition *g*(**k**) = −*g*(**k**′) at a maximum number of momenta to yield the largest positive pairing strength *λ*. Since the dominant peak in *χ*(**q**, 0) is determined by the FS scattering enhancement (or equivalently FS nesting) due to spin fluctuations, the relative strength of pairing symmetry with respect to others depends only on the strength of FS nesting, and is very much insensitive to the specific values of 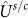. Therefore, we perform the pairing strength calculation for a realistic range of interaction values and draw conclusions based on the robust result from the overall phase diagrams. Based on the prior knowledge from other systems^1^,^9^, we know that the intra-band interaction, denoted by *U*^2^*χ_nn_*, favors *d*-wave pairing while the inter-band interaction, *V*^2^*χ_nm_*_(≠*n*)_, enhances the *s*^±^-wave pairing, where *χ_nn_* and *χ_nm_*_(≠*n*)_ are intra- and inter-band susceptibilities with *χ_nm_*_(≠*n*)_ > *χ_nn_* in these compounds. Figure 3g–l show the total pairing strength *λ* for *d*-wave (left column) and *s*^±^-wave pairing (right column) for three Pu-based superconductors (in three horizontal rows) as a function of *U* and *V*. In the entire phase diagram, we therefore predict that the *s*^±^-wave pairing dominates over the *d*-wave pairing for all three materials by as large as an order of magnitude. In addition, we also observe that the value of *λ* is maximum in PuCoGa_5_, which is consistent with its measured value of SC transition temperature *T_c_*.

**Figure 4:**
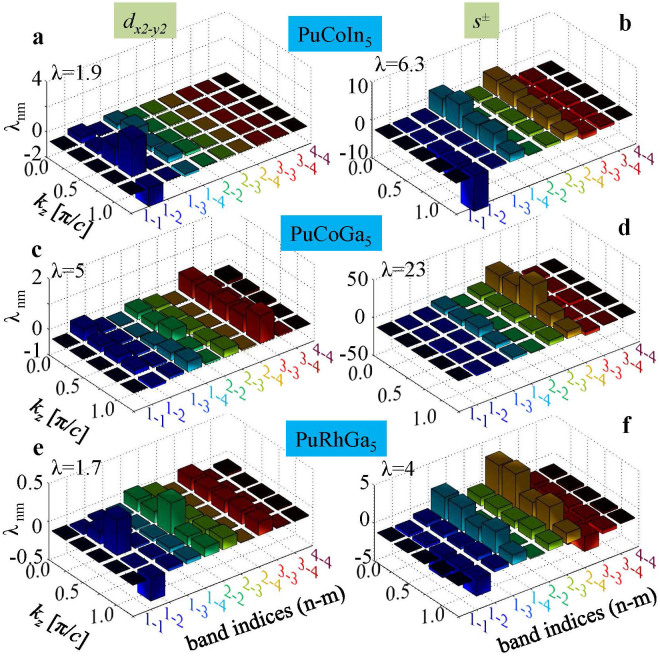
Band-dependent SC pairing strength for several representative *q_z_* cuts. A comprehensive view of band-specific pairing eigenvalues *λ_nm_* for fixed Coulomb interactions *U* = *V* = 0.5 eV in all bands. In agreement with FS topologies and hot-spots, the inter-band pairing between bands 1,2 to 3,4 contributes a large value to the *s*^±^-wave pairing. For the 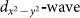 pairing, all bands contribute finite values with stronger contributions coming from *λ*_22_ and *λ*_33_, etc.

To further delineate the reasons for having a strong *s*^±^-wave pairing channel, we investigate the contributions of each band and the hot-spot wavevector in Fig. 4. We recall the relevant facts pertaining to the FS topology discussed in Fig. 1 that the dominant FS instability, which also induces the leading pairing instability, commences between bands 1 and 2 to bands 3 and 4 [connected by wavevector *Q* ~ (*π*, *π*, *q_z_*)]. The sign reversal of the pairing states, which is essential for yielding positive *λ*, occurs only in the inter-band components between bands 1,2 and bands 3,4 for the *s*^±^-wave pairing symmetry (not for intra-band except band 2 or between bands 1 and 2, or 3 and 4), while for the *d*-wave pairing symmetry both intra- and inter-band components contribute. These facts manifest themselves in the value of *λ_nm_*(*q_z_*), plotted in Fig. 4. For the *s*^±^-wave pairing, finite positive pairing strength thus occurs for *λ*_13_, *λ*_23_, *λ*_14_, and *λ*_24_, while the others contribute negative or small pairing strength. Because these pairing components are supported by the strong FS scattering enhancements, the total pairing strength *λ* eventually gains a positive and large value. This scenario should be contrasted with the *d*-wave pairing, in which the total *λ* is positive but smaller in amplitude lest all components of *λ_nm_* (except *λ*_12_ in the *q_z_* ~ *π*-plane) contributing positive values. This is due to the fact that the *d*-wave pairing, breaking *C*_4_ symmetry, obtains sign reversal in each quadrant of the Brillouin zone, thus the momentum sum in Eq. (3) amounts to a lower amplitude in *λ*. On the other hand, the *s*^±^-wave pairing channel causes fairly isotropic and single-sign pairing in each band (except in band 2, which cuts through the nodal plane), and thus contributes large phase space of positive values in the momentum sum. These are the key reasons why the nodal *s*^±^-wave pairing is favored over the *d*-wave pairing channel, although both pairing symmetries attain positive values, and are possible contenders for unconventional superconductivity in the Pu-115 family. As mentioned earlier, the 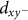 or 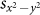-wave pairing symmetries also obtain positive *λ*, but much weaker in strength and thus are losing contenders in these systems.

### Magnetic Resonance Mode and Signatures of Pairing Symmetry

We present experimentally verifiable signatures for both dominant pairing symmetries. In this context, the magnetic resonance mode is widely considered to be a deciding feature for unconventional pairing symmetry. A well defined spin resonance is observed in cuprates^34^, iron pnictides^35^, and Ce-based heavy fermions^36^, which is located at characteristic energy and momentum in the SC state. It was shown by Yu *et al.*^2^ that the resonance energy scales almost linearly with the SC gap in all these materials, suggesting further that the magnetic resonance mode is indeed a feedback effect of the unconventional gap symmetry. Motivated by this universal scaling, we study the evolution of the magnetic excitation spectrum of both SC states and evaluate their characteristics to guide experimental detection.

**Figure 5:**
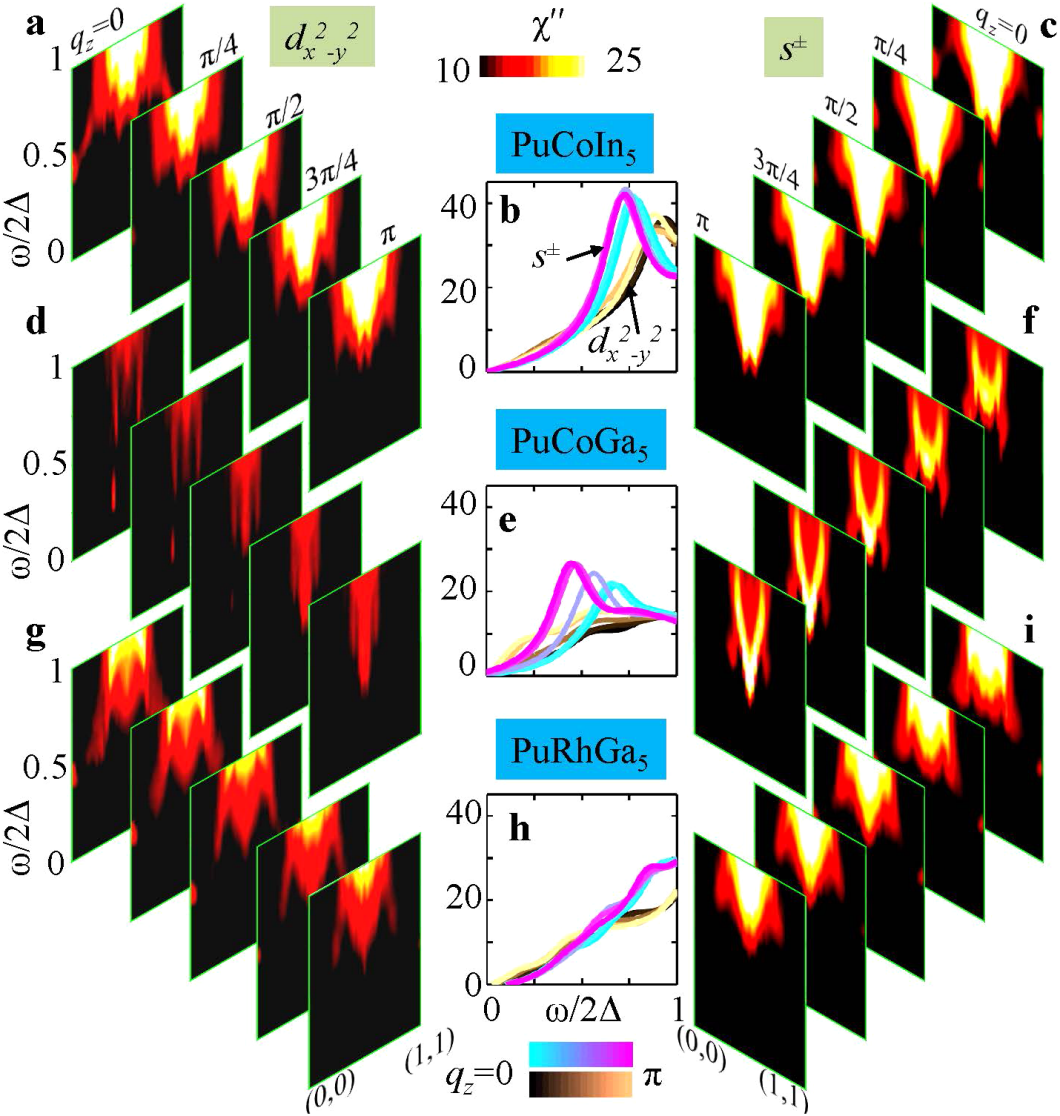
Evolution of computed magnetic excitation spectra for both pairing symmetries (left and right columns) and all three compounds (rows). Each slice of the colormap images shows the imaginary part of the BCS susceptibility within the RPA method, Im*χ*_BCS-RPA_, along wavevector **q** = (0, 0, *q_z_*) → (*π*, *π*, *q_z_*), with different slices for different *q_z_* values. The middle panel plots a single cut **Q** = (*π*, *π*, *q_z_*) as a function of excitation energy *ω* to visualize the feature of the resonance mode at an energy scale *ω* < 2Δ, where Δ is the SC gap amplitude. We set *U* = *V* = 0.4 eV for the resonance calculation.

The magnetic resonance spectrum in the SC state of single-band and multiband systems is well studied within the BCS theory^28^,^29^,^30^,^31^,^32^,^33^. A generalization to the DFT band structure is obtained here (see supplementary material for details). The magnetic collective mode is a manifestation of many-body interactions, which is captured within the BCS-RPA framework. In this framework, the RPA formulas remain the same as before, while the bare susceptibility is replaced by the BCS susceptibility which involves additional terms coming from particle-particle, and hole-hole scattering process. The magnetic resonance calculation uses the full BCS-RPA susceptibility as shown in Fig. 5. However, to obtain a qualitative understanding of the fundamental energy and momentum scale of the resonance, one can use a simplified expression to estimate the resonance condition 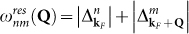, given that 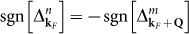, where **k***_F_* are those Fermi hot-spots, which provide strong nesting for wavevector **Q**. Such an analysis has been successfully used before for cuprates^37^ and pnictides^38^, with its quantitative value and intensity subject to the details of the band structure and the orbital overlap of matrix-element parameters.

Clearly, the condition for having a strong resonance mode has the same underlying mechanism as that of the positive pairing strength discussed earlier. Consistent with the afore-mentioned discussion, we thus expect to have spin resonance in the vicinity of **Q** = (*π*, *π*, *q_z_*), which involves a sign reversal in both *d*-wave and *s*^±^-wave pairing symmetries. Our results of magnetic resonance spectra are shown in Fig. 5 for both pairing symmetries. As for the value of *λ* in both cases, the intensity of the magnetic excitation spectrum is weaker and more spread out over the momentum space for the *d*-wave pairing case, while it is substantially more localized around **Q** with maximum intensity shifted towards *q_z_* → *π*/*c* for the *s*^±^-wave pairing case. To affirm our statement, in Fig. 5 we plot the total *χ*(**Q**, *ω*) (the energy axis is normalized by the SC gap amplitude to perform a comparative study between different materials with different *T_c_*) along the diagonal direction and at five representative *q_z_* cuts. Also the single-momentum cuts at (*π*, *π*, *q_z_*) are plotted for both pairing symmetries with different colormaps distinguishing different *q_z_* values in the middle column, while different rows are for different materials. We clearly see the so-called localized collective mode for the *s*^±^-wave pairing in both PuCoIn_5_, PuCoGa_5_, but not in PuRhGa_5_ system. Our prediction of the ratio *ω_res_*/2Δ ~ 0.5–0.75 is in reasonable agreement with universal scaling^2^ and can be verified by inelastic neutron scattering measurements, which has the ability to detect both the energy and momentum resolved collective **S** = 1 spin excitations.

### PCS Results

**Figure 6:**
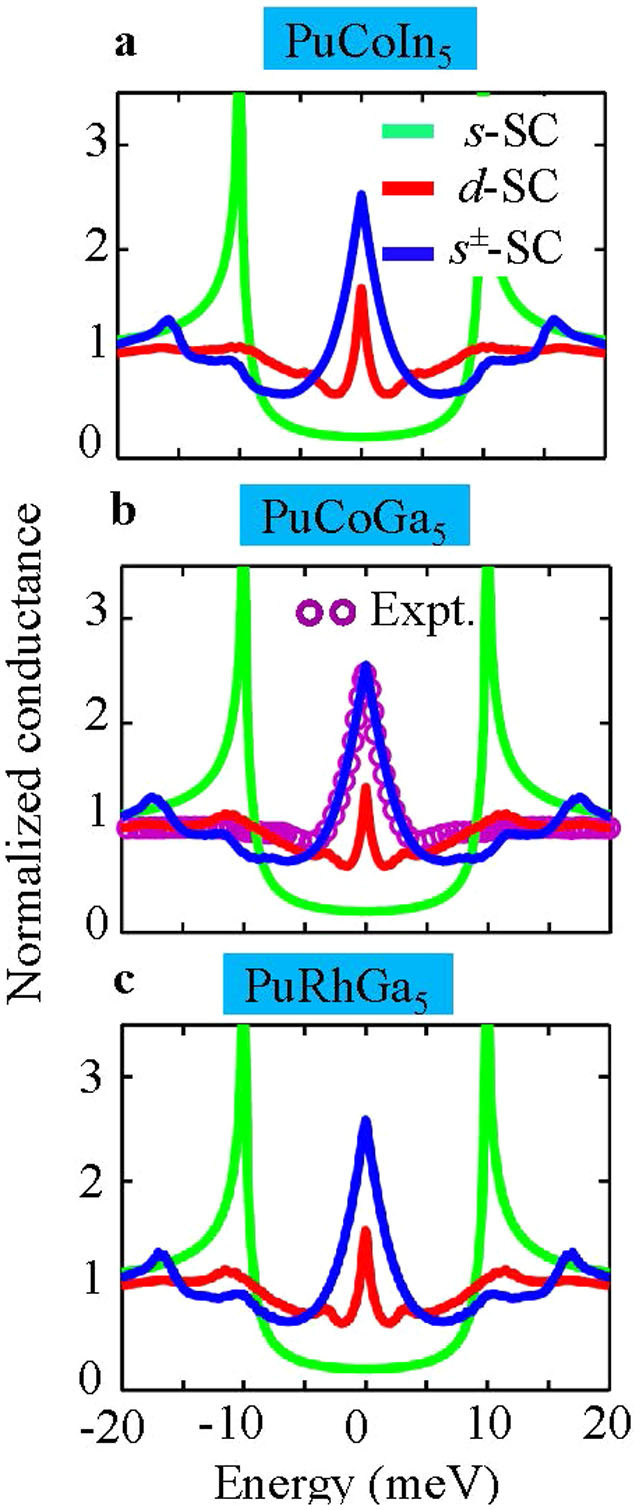
PCS conductances. Computed PCS spectra for nodal 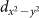-(red), *s*^±^-(blue), and isotropic *s*^++^ -wave (green) gaps using a generalized multiband BTK formalism. All data are normalized to their normal-state conductance. A zero-bias conductance peak is seen in both nodal 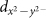 and *s*^±^-wave gaps, but not in the fully gapped isotropic *s*-wave gap.

To elaborate on the spectroscopic fingerprints of both pairing symmetries, we calculate their respective PCS spectra using the Blonder-Tinkham-Klapwijk (BTK) formalism^39^ generalized to multiband systems with anisotropic FSs and SC order parameters^40^,^41^, see supplementary materials. In order to keep the problem tractable, we consider only normal incidence of electrons from the metallic tip and neglect interband transitions as well as Fermi velocity mismatch between the tip and the Pu-115 compounds. The corresponding results are given in Fig. 6 and compared with available conductance data for PuCoGa_5_^19^ in Fig. 6b. We see that for both nodal *d*-wave and *s*^±^-wave pairing symmetries, the calculated conductance spectrum exhibits a characteristic zero-bias conductance peak, which marks the presence of nodes and the hallmark of Andreev bound states. To contrast these results, we also calculate the PCS spectrum for isotropic (nodeless) *s*-wave pairing (green line) which shows a suppressed Andreev reflection signal for finite interface barrier potential. For a reasonable parameter choice of Δ_0_ = 10 meV, interface transparency coefficient *Z* = 1.55, and a rotation angle *α* = *π*/8 (of the crystallographic *a* axis with respect to the normal of the interface), we can fit the experimental data very well with nodal *s*^±^-wave pairing symmetry. Of course, it is not impossible to fit the data with *d*-wave with another parameter choice even in this realistic multiband model. This implies that PCS conductance data are consistent with nodal gap functions, but cannot unequivocally determine the locations of nodes.

### Outlook

Obtaining a consistent theory of unconventional superconductivity, which can describe cuprate, pnictide, organic, heavy-fermion, as well as actinide superconductors has a pressing need. Considerable consistency is achieved so far in all three former families of superconductors^1^,^9^,^28^,^29^,^30^,^31^,^32^,^33^ in terms of spin-fluctuation-mediated superconductivity, pairing symmetry, and magnetic resonance mode, which motivated us to perform these studies in the actinide family. Here we provided the first DFT-based spin-fluctuation calculation of the pair symmetry in the three-dimensional, multiband actinide superconductor family and find the surprising result of the dominant *s*^±^-wave pairing symmetry with a nodal gap, and not the so often assumed *d*-wave gap. The feedback effect of this unconventional pairing yields a strong magnetic resonance mode, which can be tested in future inelastic neutron scattering measurements. In the past, the *d*-wave gap was mostly proposed because it was the simplest scenario based on a single band that could explain power laws in the low-temperature behavior of specific heat, spin-lattice-relaxation rate, and magnetic penetration depth. Of course, gap nodes on the FS have a profound influence on electronic excitations and the formation of Andreev bound states, which provide a natural explanation of the observed zero-bias conductance peak in the point-contact spectra^41^,^42^,^43^. Interestingly, the observed zero-bias conductance peak can be fit equally well with the nodal *s*^±^-wave pairing symmetry as shown here. The identification of the nodal *s*^±^-wave pairing symmetry will also provide insight into the physics of iron-pnictide superconductors, which are believed to host unconventional pairing symmetry. Therefore, we envisage that further studies of this actinide family of unconventional multiorbital superconductors will advance the lofty goal of obtaining a unified spin-fluctuation picture of superconductivity.

## Author Contributions

T.D. has performed major calculations, analysis, and written up the paper. J.-X.Z. has performed the first-principles band structure calculation and contributed to the discussions of results. M.J.G. has contributed to the calculation of point contact spectroscopy, and in the analysis and writing. All authors have participated in the revision of the manuscript.

## Supplementary Material

Supplementary Information Supplementary Material
