# Supplementary material for: Theory of nodal s±-wave pairing symmetry in the Pu-based 115 superconductor family
Source: Sci Rep. 2015 Feb 27;5:8632. doi: 10.1038/srep08632 (PMC4342998; doi:10.1038/srep08632)
Supplement: Supplementary Information — Supplementary Material [file srep08632-s1.pdf]

Supplementary Material for "Theory of nodal $s^{\pm}$-pairing symmetry in the Pu-based 115 superconductor family"

Tanmoy Das[1], Jian-Xin Zhu[1,2], & Matthias J. Graf[1]

[1]Theoretical Division, Los Alamos National Laboratory, Los Alamos, NM 87545, USA [2]Center for Nanotechnology, Los Alamos National Laboratory, Los Alamos, NM 87545, USA.

(Dated: November 5, 2014)

I. DENSITY FUNCTIONAL THEORY CALCULATIONS.

We have performed electronic structure calculations of these materials within the framework of DFT and the results are shown in Fig. 1 of the main text. Our calculations were carried out by using the full-potential linearized augmented plane wave (FP-LAPW) method as implemented in the WIEN2k code [1]. The generalized gradient approximation (GGA) [2] was used for the exchange-correlation functional. The spin-orbit coupling was included in a second variational way, for which relativistic $p_{1/2}$ local orbitals were added into the basis set for the description of the $6p$ states of plutonium [3]. The muffin-tin radii were: $2.5a_0$ for Pu, $2.46a_0$ ($PuCoGa_5$) and $2.5a_0$ ($PuCoIn_5$) for Co, $2.5a_0$ for Rh, $2.18a_0$ ($PuCoGa_5$) and $2.23a_0$ ($PuRhGa_5$) for Ga, and $2.39a_0$ for In, where a_0 is the Bohr radius. The energy spread to separate the localized valence states was -6 Ryd. The criterion for the number of plane waves was $R^{\min}_{MT}K^{\max} = 8$ and the number of $\mathbf{k}$-points was $40 \times 40 \times 25$. The experimentally determined crystallographic structures were used [4–6].

II. POINT-CONTACT SPECTROSCOPY CALCULATION USING ANISOTROPIC FERMI SURFACES AND ANISOTROPIC ORDER PARAMETER

We calculate the point-contact spectrum (PCS) by taking into account the full Fermi surface (FS) anisotropy of a multiband system with anisotropic order parameter by following the formalism given in Refs. [7–10]. For simplification, however, we only include the anisotropy in the FS of the superconducting (SC) material, while that of the normal metal tip is neglected. Let us define $\mathbf{n}$ as the unit vector in the direction of the total injected current, which for simplicity we choose to be perpendicular to the contact interface between the SC (S) and normal metal (N) interface. As a consequence the components along the direction $\mathbf{n}$ of the Fermi velocities at wavevector $\mathbf{k}$ in the ith FS sheet of the superconductor are $\mathbf{v}_{i\mathbf{k}} \cdot \mathbf{n} = v_{i\mathbf{k},n}$, where $\mathbf{v}_{i\mathbf{k}} = -\frac{1}{\hbar}\nabla_{\mathbf{k}} E_{i\mathbf{k}}$, and $E_{i\mathbf{k}}$ is the corresponding quasiparticle band. Generalizing the Blonder, Tinkham and Klapwijk (BTK) formula [11] to anisotropic FSs of multiband superconductors, it was shown that the total normalized conductance seen along the direction $\mathbf{n}$ can be written as [10]

$$\langle G(E)\rangle_{I\|\mathbf{n}} = \frac{\sum_i \langle \sigma_{i\mathbf{k}}(E) D_{i\mathbf{k}} v_{i\mathbf{k},n}\rangle_{\mathrm{FS}_i}}{\sum_i \langle D_{i\mathbf{k}} v_{i\mathbf{k},n}\rangle_{\mathrm{FS}_i}}, \tag{1}$$

where $D_{i\mathbf{k}} = 1/v_{i\mathbf{k}}$ is the density of states on the ith FS sheet, and $\sigma_{i\mathbf{k}}(E)$ is the BTK SC transition probability calculated as follows. Here we neglect interband interference effects and variations in the tunneling matrix elements (different weight factors) between the normal tip and different bands of the superconductor.

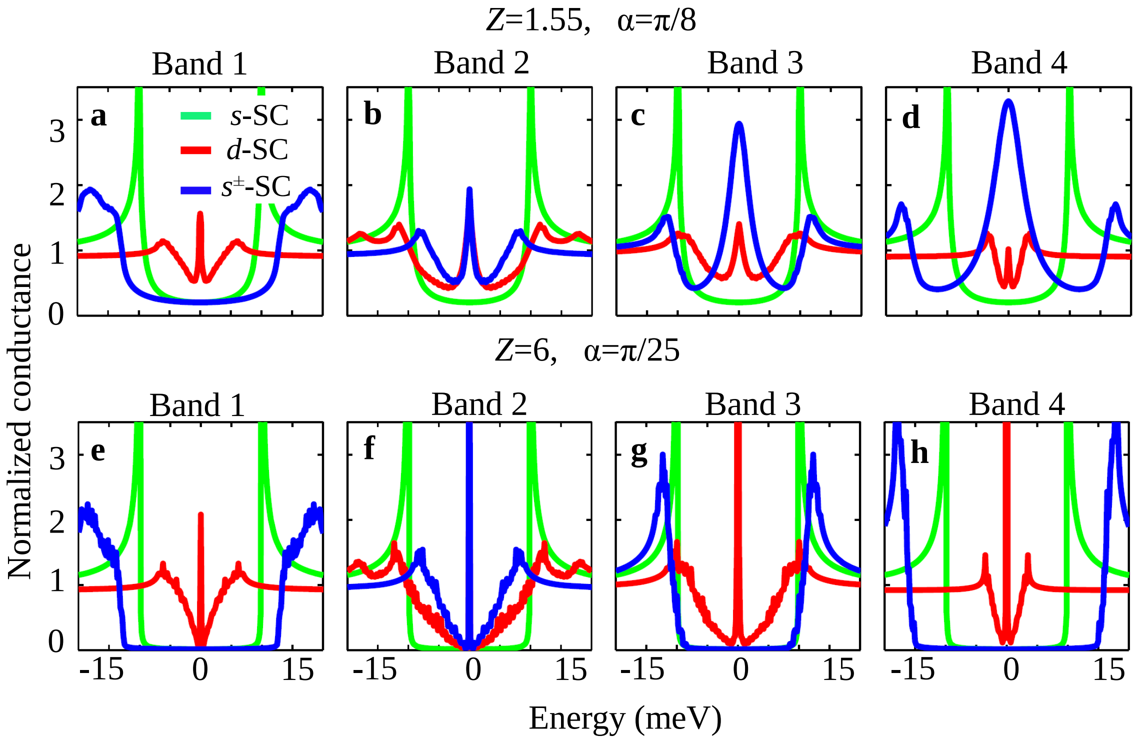

Fig. S1: Calculated PCS spectra, decomposed in different bands for two parameter sets for $PuCoGa_5$. The zero-bias conductance peak is present in all bands for d-wave pairing and in band 2 for $s^{\pm}$-pairing as expected. For a larger value of α, we find that a zero-bias conductance peak is induced in bands 3 and 4 in $s^{\pm}$-wave pairing, in addition to band 2. Since band 3 and 4 have larger gap amplitudes which enables a broader PCS conductance peak and match the experimental data, as shown in (a)-(d). For $\alpha = \pi/25$, the conductance peak only survives in band 2 for this pairing.

Let us assume that $\theta_{i\mathbf{k}}$ is the transmission angle at the interface between the normal and SC materials for band i at Fermi momentum $\mathbf{k}$. The specific SC gap function is defined in terms of $\theta_{i\mathbf{k}}$ as $\Delta_{i\mathbf{k}}(\theta_{i\mathbf{k}}) = \Delta_0(\cos k_x^i \pm \cos k_y^i)$, for $s^{\pm}$- and d-wave pairing, respectively and $k_{x,y}^i$ are the Fermi momentum for the ith-band. Then $\theta_{i\mathbf{k}} = \tan^{-1}(k_y^i/k_x^i)$. Lets us also define α as the rotation of the crystallographic a-axis with respect to the normal to the interface (x axis). In this circumstance, the electron-like and hole-like quasiparticle (EQs/HQs) injected in the SC material with angles $\pm\theta_{i\mathbf{k}}$, they access different gap values as $\Delta_{i\mathbf{k}}^{\pm} = \Delta_{i\mathbf{k}}(\pm\theta_{i\mathbf{k}} - \alpha)$. In this case the SC transition probability becomes

$$\sigma_{j\mathbf{k}}(E) = \tau_{\rm N}\frac{1 + \tau_{\rm N} \mid \gamma_{j\mathbf{k}}^{+}(E) \mid^2 + (1-\tau_{\rm N}) \mid \gamma_{j\mathbf{k}}^{+}(E)\gamma_{j\mathbf{k}}^{-}(E) \mid^2}{\mid 1 + (1-\tau_{\rm N}) \mid \gamma_{j\mathbf{k}}^{+}(E)\gamma_{j\mathbf{k}}^{-}(E) \exp{(i\phi_{i\mathbf{k}})} \mid^2}, \tag{2}$$

where the function

$$\gamma_{i\mathbf{k}}^{\pm}(E) = \frac{\mid E \mid - \sqrt{E^2 - \mid \Delta_{i\mathbf{k}}^{\pm} \mid^2}}{\mid \Delta_{i\mathbf{k}}^{\pm} \mid}, \tag{3}$$

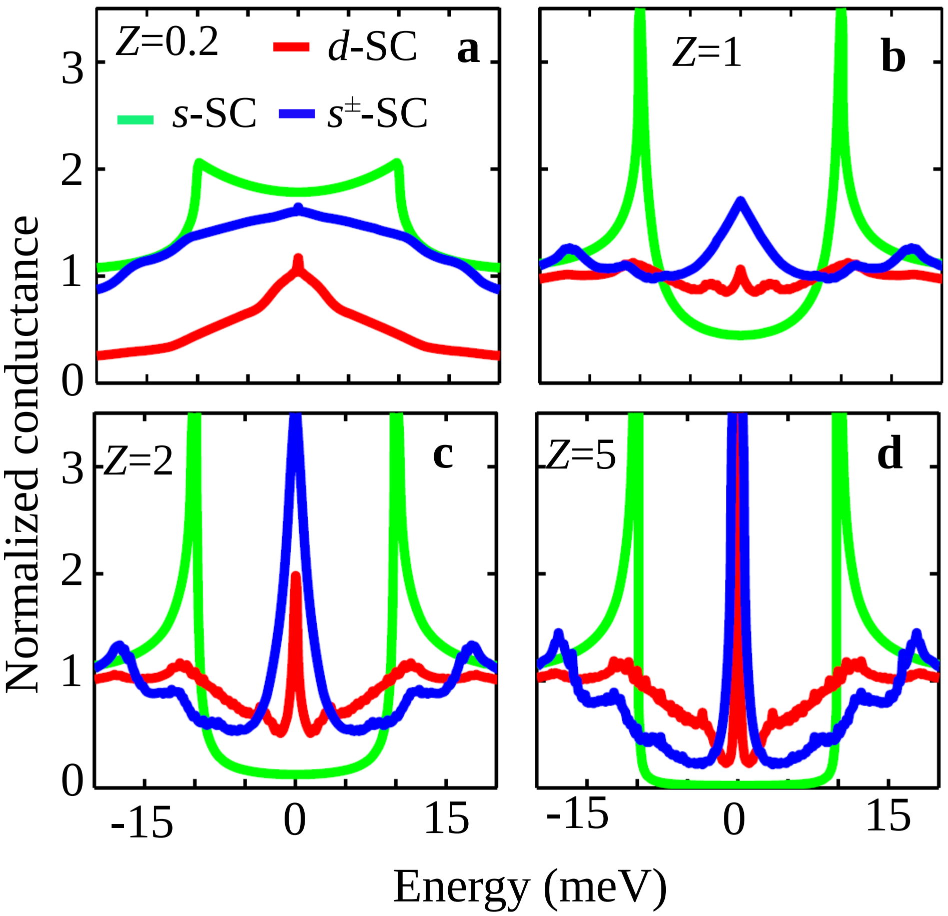

Fig. S2: Calculated PCS spectra at different parameters of the interface barrier potential Z with $\alpha = \pi/8$, and Δ_0=10 meV.

and $\phi_{i\mathbf{k}} = \phi^-_{i\mathbf{k}} - \phi^+_{i\mathbf{k}}$, with $\phi^\pm_{i\mathbf{k}}$ being the phases of $\Delta^\pm_{i\mathbf{k}}$. It should be noted that $\gamma^\pm_{i\mathbf{k}}(E)$ are, in general, complex functions even if the momentum-dependent gap $\Delta_{i\mathbf{k}}$ is real when $E < \Delta_{i\mathbf{k}}$. For simplicity, we further assume a band-independent and momentum-independent interface barrier, the parameter τ_N is the transparency factor of the barrier in the BTK approximation of current injection perpendicular to the SN interface, defined as $\tau_\mathrm{N} = 1/(1+Z^2)$. The limit $Z = 0$ gives the perfectly transparent junction in the transmission limit, i.e., the ideal Andreev reflection regime.

In Fig. 6 of the main text, we presented results of the PCS conductance for the nodal $d_{x^2-y^2}$-wave, nodal $s^\pm$-wave pairing symmetries, and compared these results with a nodeless and fully isotropic s-wave pairing symmetry. For the best fit to the available experimental data of $PuCoGa_5$,[12], we set $Z = 1.55$, $\alpha = \pi/8$ and a fixed SC gap amplitude of $\Delta_0 = 10$ meV for all three pairing symmetries. We showed that a single and sharp zero-bias peak is reproduced for $s^\pm$-pairing. Of course, even in this multiband setup, it may be possible to fit the experimental

data with d-wave pairing.

In Fig. S1, we showed the band decomposed PCS spectra for two different parameter sets for three different parameter sets. The evolution of the PCS spectrum for different values of Z is discussed in Fig. S2.

III. MULTIBAND COULOMB INTERACTIONS

For intermetallic actinides, the spin-orbit coupling is very strong and causes a band splitting of the $5f$ states of about 1 eV. This quenches the Hund's coupling term, because $J_H \ll \lambda_{SOC}$. The remaining interaction terms account for the onsite intra- and inter-band Coulomb repulsions as given by the interaction Hamiltonian

$$H_{int} = \sum_{\mathbf{k},\mathbf{k}'}\left[\sum_n U_n c^{n\dagger}_{\mathbf{k}\uparrow}c^{n}_{\mathbf{k}\uparrow}c^{n\dagger}_{\mathbf{k}'\downarrow}c^{n}_{\mathbf{k}'\downarrow} + \sum_{n\neq m,\sigma,\sigma'} V_{nm}c^{n\dagger}_{\mathbf{k}\sigma}c^{n}_{\mathbf{k}\sigma}c^{m\dagger}_{\mathbf{k}'\sigma'}c^{m}_{\mathbf{k}'\sigma'}\right]. \quad (4)$$

Here $c^{n\dagger}_{\mathbf{k}\sigma}(c^{n}_{\mathbf{k}\sigma})$ creates (annihilates) a Bloch state at momentum $\mathbf{k}$, (pseudo-) spin $\sigma = \uparrow / \downarrow$ in the nth-band. The interaction matrices $\tilde{U}^{s/c}$ used in the RPA formalism consist of components U_n and V_{nm}. The different bandwidths of different bands amount to different critical values of U and V, determined by the positive RPA denominator as $\tilde{U}^{s/c}_{nm}\tilde{\chi}_{nm} \leq 1$. Using this condition we find that the critical interaction values for bands 2 and 3 are considerably small and we fix these values to be $2U_2 = V_{13} = V_{23} = 200$ meV at all points for the calculations of λ in Fig. 3. The rest of the interactions are taken to be same for all bands as $U_n = U$, and $V_{nm} = V$. In drawing the parameter space in Fig. 3, we limit the values of U and V to below 600 meV and 700 meV, respectively, because for larger values the RPA susceptibilities for bands 3 and 4 become negative, which is above the critical values set by the RPA denominator. However the general conclusion of the dominant $s^{\pm}$-wave pairing than d-wave pairing symmetry strength λ is consistent throughout the $U-V$ map. Since the essential physics is determined by the FS topology and nesting conditions, we anticipate that this conclusion also remains valid for higher values of U and V.

IV. BCS SUSCEPTIBILITY AND SPIN RESONANCE

In this section, we give the details of our spin susceptibility calculation presented in the main text. All "super" matrices with tilde are defined as $\tilde{\chi}_{ij} = \chi_{nm}\delta_{ij}$, etc., with super indices $i = 4(n-1)+m$, and $n, m = 1-4$ are band indices. Thus variables with tilde have matrix dimension 16×16. We follow closely earlier work [17, 19] and evaluate the spin-resonance susceptibility in

the SC state within the random phase approximation (RPA) of the BCS formalism, which is given by the bare bubble transverse spin susceptibility

$$\chi_{nm}(\mathbf{q},\omega) = \int \frac{d\mathbf{k}}{\Omega_{\rm BZ}}\, M_{nm}(\mathbf{k},\mathbf{q}) \left\{ \frac{1}{2}\left[1 + \frac{\xi^n_{\mathbf{k}}\xi^m_{\mathbf{k+q}} + \Delta^n_{\mathbf{k}}\Delta^m_{\mathbf{k+q}}}{E^n_{\mathbf{k}}E^m_{\mathbf{k+q}}}\right] \frac{f(E^n_{\mathbf{k}}) - f(E^m_{\mathbf{k+q}})}{\omega - E^n_{\mathbf{k}} + E^m_{\mathbf{k+q}} + i\delta} \right.$$
$$+ \frac{1}{4}\left[1 + \frac{\xi^n_{\mathbf{k}}}{E^n_{\mathbf{k}}} - \frac{\xi^m_{\mathbf{k+q}}}{E^m_{\mathbf{k+q}}} - \frac{\xi^n_{\mathbf{k}}\xi^m_{\mathbf{k+q}} + \Delta^n_{\mathbf{k}}\Delta^m_{\mathbf{k+q}}}{E^n_{\mathbf{k}}E^m_{\mathbf{k+q}}}\right] \frac{1 - f(E^n_{\mathbf{k}}) - f(E^m_{\mathbf{k+q}})}{\omega + E^n_{\mathbf{k}} + E^m_{\mathbf{k+q}} + i\delta}$$
$$\left. + \frac{1}{4}\left[1 - \frac{\xi^n_{\mathbf{k}}}{E^n_{\mathbf{k}}} + \frac{\xi^m_{\mathbf{k+q}}}{E^m_{\mathbf{k+q}}} - \frac{\xi^n_{\mathbf{k}}\xi^m_{\mathbf{k+q}} + \Delta^n_{\mathbf{k}}\Delta^m_{\mathbf{k+q}}}{E^n_{\mathbf{k}}E^m_{\mathbf{k+q}}}\right] \frac{f(E^n_{\mathbf{k}}) + f(E^m_{\mathbf{k+q}}) - 1}{\omega - E^n_{\mathbf{k}} - E^m_{\mathbf{k+q}} + i\delta} \right\} \tag{5}$$

and the RPA susceptibility is attained with the onsite Coulomb interaction super matrix, $\tilde{U}$,

$$\tilde{\chi}_{RPA}(\mathbf{q},\omega) = \left[1 - \tilde{U}\tilde{\chi}(\mathbf{q},\omega)\right]^{-1} \tilde{\chi}(\mathbf{q},\omega). \tag{6}$$

Here $E^n_{\mathbf{k}} = [(\xi^n_{\mathbf{k}})^2 + (\Delta^n_{\mathbf{k}})^2]^{1/2}$ is the SC quasiparticle energy of the eigenstate $\xi^n_{\mathbf{k}}$ (n is the band index) and $\Delta^n_{\mathbf{k}}$ is the SC gap function. $M_{nm}(\mathbf{k},\mathbf{q})$ is the matrix element consisting of the eigenstates of the initial and final scattered quasiparticle states. In Eq. 5, the first term is called particle-hole scattering term, which vanishes for $\omega \leq 2\Delta$, regardless of the pairing symmetry due to particle-hole symmetry. The second and third terms are for particle-particle and hole-hole scattering, respectively, which become active in the SC state. Focusing on the third term (a similar analysis applies to the second term), we find that this term contributes a non-zero value only when sign$[\Delta^n_{\mathbf{k}}] \neq$sign$[\Delta^m_{\mathbf{k+q}}]$ (since $\xi^n_{\mathbf{k}} = 0$ on the Fermi surface). A pole is thus obtained in the imaginary part of χ_{nm} at

$$\omega^{\rm res}_{nm}(\boldsymbol{q}) = |\Delta^n_{\mathbf{k}}| + |\Delta^m_{\mathbf{k+q}}|. \tag{7}$$

Of course, the many-body and matrix-element effects can shift the energy scale as discussed in the Method section in the main text.

III. COMPUTED GAP FUNCTION AND WEAK HIGHER-ORDER HARMONICS

In the main text, we calculated the pairing eigenfunction $g(\mathbf{k})$ by directly solving the eigenvalue problem, which is obtained by rewriting the linearized weak-coupling multiband gap equation. We also calculated the so-called pairing strength λ in two different, yet equivalent, procedures by using Eqs. (3) and (4). We calculated the maximum eigenvalue λ and corresponding eigenfunction $g(\mathbf{k})$ by solving the eigenvalue matrix problem for the spin-fluctuation pairing vertex, in Eq. (3). This involves summation over the 3D FSs of all 4 bands. For each band we expand the Γ_{nm} matrix

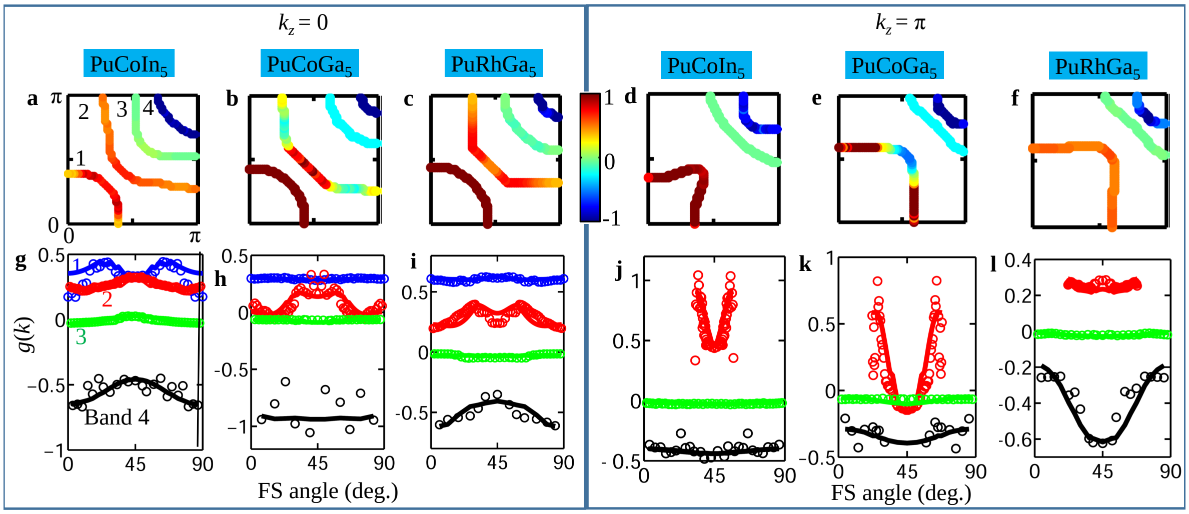

Fig. S3: Top panel: Computed k-dependent SC gap function plotted in colormap as in the main text. Bottom panel: The same gap function of four bands plotted as a function of FS angle. The solid lines are the fit with the gap function $g(k)$ including higher harmonics as given in Eq. (11), and the relevant parameters are given in Table I. The FS angle is defined to be zero along the zone boundary direction (100) for band 1, and (010) for all other bands, and 45° along the diagonal direction (110) for all bands.

into discretized Fermi momenta $\mathbf{k}_n$ and $\mathbf{k}'_m$. In our case, since the Γ_{nm} matrix is defined in the band basis, each matrix index n assumes dimension N_n (number of points for the nth-band), with a total number of $N = \sum_{n=1}^{4} N_n$ FS momenta, where N_n is the number of 3D Fermi momenta for the nth band. Therefore the matrix we diagonalize has a dimension of $N \times N$.

The maximum eigenvalue is proportional to the highest superconducting transition temperature and the corresponding eigenvector gives the leading pairing function $g(\mathbf{k})$, which is plotted in Fig. 3 (left-hand side) and in Fig S3. The first point to notice in Fig. S3 is that there is no clear fourfold symmetry breaking in $g(\mathbf{k})$, and that there is no gap node along the diagonal direction. This finding clearly excludes the presence of any significant d-wave pairing component. In a final step, we analyze the pairing function in more detail by fitting it up to third harmonics of $s^{\pm}$-pairing symmetry [19]:

$$g(\mathbf{k}) = a_1(\cos k_x + \cos k_y) + 2a_2 \cos 2k_x \cos 2k_y + 2a_3 \cos 4k_x \cos 4k_y + a_0. \tag{8}$$

The corresponding fits are shown in Fig. S3. The general conclusion drawn from these fits is that the coefficients for second and third harmonics a_2 and a_3, respectively, are almost an order of magnitude lower than the first harmonic of $s^{\pm}$- pairing. We also notice a weak k_z dependence in the fit parameters. Furthermore, the result shows that the gap anisotropy is largest in band 2.

Table. I: Coefficients of various harmonics of the gap function given in Eq. (11).

	Band 1		Band 2		Band 3		Band 4	
$PuCoIn_5$	$k_z = 0$	$k_z = \pi$	$k_z = 0$	$k_z = \pi$	$k_z = 0$	$k_z = \pi$	$k_z = 0$	$k_z = \pi$
a_1	0.35	–	0.15	0.5	0.12	0.12	0.3	0.2
a_2	0.06	–	0.025	0.05	0.015	0.015	-0.06	-0.02
a_3	0.1	–	0	0.05	0	0	-0.06	-0.02
a_0	0.9	–	0.25	0.21	0.1	0.1	0.1	-0.08
$PuCoGa_5$								
a_1	0.13	–	0.35	0.7	0.1	0.1	0.25	0.25
a_2	-0.013	–	-0.02	-0.07	-0.01	-0.01	-0.025	-0.025
a_3	0	–	-0.02	-0.07	-0.01	-0.01	-0.05	0.05
a_0	0.15	–	0.05	0.1	0.08	0.08	-0.4	0.1
$PuRhGa_5$								
a_1	0.18	–	0.35	0.1	0.1	0.05	0.2	0.17
a_2	-0.01	–	-0.087	0.07	-0.03	0	-0.05	-0.17
a_3	-0.013	–	-0.025	0.025	0	0	0.2	-0.2
a_0	0.8	–	0.27	0.24	0.11	0.05	0.05	0

This is where the nodes are located. The gap amplitude is smallest in band 3 and then it increases from band 2 to band 1 to band 4. This is expected from the $s^{\pm}$-pairing symmetry as the gap maxima lie at the Γ and M points, with opposite sign. Therefore, our conclusion about the $s^{\pm}$ pairing symmetry in the Pu-based superconductor is a robust feature.

Secondly, we calculated the pairing strength through the usual projection of the eigenvalue Eq. (3) onto selected orthogonal pairing functions with characteristic symmetry. The projected pairing strength for a given pairing symmetry g_α is calculated from Eq. (4) of the main text, which was used earlier in Refs. [18, 19]. Finally, the total pairing strength is obtained by summing over all indices, $\lambda^\alpha = \sum_{n,m} \lambda^\alpha_{nm}$, and is plotted as a function of the Coulomb potentials U and V in Fig. 3 (right-hand side). The line integrals over each FS sheet were performed over FS pockets in each corresponding k_z plane, and then summed over k_z slices. The intra- and interband pairing strength λ^α_{nm} is plotted in Fig. 4 as a function of the k_z slices.

[1] Blaha P. *et al.*, *An augmented plane wave + local orbitals program for calculating crystal properties*, (K. Schwarz, Tech. Universität Wien, Austria, 2001).

[2] Perdew, J. P., Burke, S., & Ernzerhof, M. *Generalized gradient approximation made simple*, Phys. Rev.

Lett. **77**, 3865 (1996).

[3] Kuneš, J., Novák, P., Schmid, R., Blaha, P., & Schwarz, K. *Electronic structure of fcc Th: Spin-orbit calculation with* $6p_{1/2}$ *local orbital extension*, Phys. Rev. B **64**, 153102 (2001).

[4] Sarrao, J. L. *et al.* Plutonium-based superconductivity with a transition temperature above 18 K. *Nature* **420**, 297 (2002).

[5] Wastin, F. *et al.* Advances in the preparation and characterization of transuranium systems. *J. Phys. Condens. Matter* **15**, S2279 (2003).

[6] Bauer, E. D. *et al.* Localized $5f$ electrons in SC $PuCoIn_5$: Consequences for superconductivity in $PuCoGa_5$. *J. Phys. Condens. Matter* **24**, 052206 (2012).

[7] Kashiwaya, S., Tanaka, Y., Koyanagi, M., & Kajimura, K. Theory for tunneling spectroscopy of anisotropic superconductors. *Phys. Rev. B* **53**, 2667-2676 (1996).

[8] Mazin I. I. How to define and calculate the degree of spin polarization in ferromagnets. *Phys. Rev. Lett.* **83**,1427 (1999).

[9] Brinkman A *et al.* Multiband model for tunneling in MgB_2 junctions. *Phys. Rev. B* **65**, 180517 (2002).

[10] Daghero, D., Gonnelli, R. S. Probing multiband superconductivity by point-contact spectroscopy. *Supercond. Sci. Technol.* **23**, 043001 (2010).

[11] Blonder G. E., Tinkham M., Klapwijk T. M., Transition from metallic to tunneling regimes in superconducting microconstrictions: Excess current, charge imbalance, and supercurrent conversion. *Phys. Rev. B* **25**, 4515 (1982).

[12] Daghero, D. *et al.* Strong-coupling d-wave superconductivity in $PuCoGa_5$ probed by point contact spectroscopy. *Nat. Commun.* **3**, 786 (2012).

[13] Carbotte, J. P. Properties of boson-exchange superconductors. *Rev. Mod. Phys.* **62**, 1027-1157 (1990).

[14] Allen P. B., Dynes R. C. Transition temperature of strong-coupled superconductors reanalyzed. *Phys. Rev. B* **12**, 905-922 (1975).

[15] Monthoux P, Balatsky A V, Pines D Toward a theory of high-temperature superconductivity in the antiferromagnetically correlated cuprate oxides. *Phys. Rev. Lett.* **67**, 3448 (1991).

[16] Schrieffer J R, Wen X G, Zhang S C Dynamic spin fluctuations and the bag mechanism of high-T_c superconductivity. *Phys. Rev. B* **39**, 11663-11679 (1989).

[17] Takimoto T, Hotta T, Ueda K, Strong-coupling theory of superconductivity in a degenerate Hubbard model. *Phys. Rev. B* **69**, 104504 (2004).

[18] Scalapino, D. J., Loh, Jr., E. & Hirsch, J. E. d-wave pairing near a spin-density-wave instability. *Phys. Rev. B* **34**, 8190 (1986).

[19] Graser S, Maier T A, Hirschfeld P J, Scalapino D J, Near-degeneracy of several pairing channels in multiorbital models for the Fe pnictides. *New J. Phys.* **11**, 025016 (2009).

[20] Yao Z-J, Li J-X, and Wang, Z D, Spin fluctuations, interband coupling and unconventional pairing in iron-based superconductors, *New J. Phys.* **11**, 025009 (2009).

[21] Das T, and Balatsky, A V, Origin of pressure induced second superconducting dome in $A_yFe_{2-x}Se_2$

[A=K, (Tl,Rb)], *New J. Phys.* **15**, 093045 (2013) .
